# Contralateral Traumatic Hemopneumothorax

**DOI:** 10.1155/2018/4328704

**Published:** 2018-12-19

**Authors:** Quevedo-Florez Leonardo Alexander, Montenegro-Apraez Alvaro Andrés, Aguiar-Martinez Leonar Giovanni, Hernández Juan Carlos, Cortés-Tascón Juan David

**Affiliations:** ^1^Fellowship Critical Care Medicine, Universidad de la Sabana, Bogotá, Colombia; ^2^Emergency Physician, Pontifical Xavierian University, Bogotá, Colombia; ^3^Internist Physician, Pontifical Xavierian University, Advanced Fellowship in Emergency Medicine, George Washington University, Emergency Medicine Postgraduate Program Director, Pontifical Xavierian University, Colombia; ^4^Internist Physician, Pneumology, Clínica de Occidente, Bogotá, Colombia; ^5^General Physician, Pontifical Xavierian University, Bogotá, Colombia

## Abstract

Pneumothorax is the entry of air into the virtual space between the visceral and the parietal pleurae, which can occur spontaneously or to a greater extent in a traumatic way. In daily clinical practice it is frequent to find injuries that generate traumatic pneumothorax that is ipsilateral to the lesion. However, there are case reports of contralateral pneumothorax that occurred in procedures such as insertion of pacemakers, or in cases of pneumonectomy. The following is the case report of a 37-year-old man who was admitted with a sharp wound to the right paravertebral region who developed a left haemopneumothorax due to a tangential course of the injuring agent. Adequate clinical judgment was followed, and several imaging studies were carried out, leading to the diagnosis of traumatic pneumothorax that was contralateral to the described injury.

## 1. Introduction

 Pneumothorax is defined as an abnormal accumulation of air or gas in the pleural space, which separates the lung from the thoracic wall [1–3]. The causes related to the production of pneumothorax are multiple and, due to this fact, they have received different denominations such as iatrogenic, traumatic, barotrauma, and spontaneous (primary and secondary) [1, 4]. Traumatic pneumothorax is produced due to a direct or indirect injury in the chest [5]; however, in some series, traumatic pneumothorax is classified depending on the cause, including iatrogenic pneumothorax and barotrauma in this classification, calling it simply penetrating and nonpenetrating pneumothorax [6]. Some of the most common causes of traumatic pneumothorax are traffic accidents, gunshot wounds, rib fractures with pulmonary perforation, and stab wounds [3, 5, 7]. Iatrogenic pneumothorax is produced by an injury of the pleura during the course of a medical procedure, either intentionally or unintentionally [8].

In this article, the main goal of the authors is to disclose that wounds caused by piercing objects in thorax do not always generate an ipsilateral trauma to the site of entrance. In many cases, the ignorance or the lack of questioning about the mechanics of the trauma, as well as the lack of medical suspicion, can cause the omission of injuries that may put the patient's life in danger. In this discussion, the importance of clinical judgment will be explained, as well as the importance of the anatomical inspection of the wounds in order to predict their path.

## 2. Clinical Case

**Figure 1:**
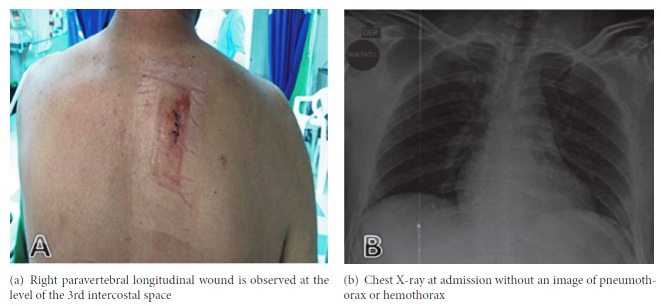


The case is about a 37-year-old patient who presented a stab wound, located in the right paravertebral region at the third intercostal space level, which was 7 cm in length (see Figure 1(a)). On admission, the patient was in good medical condition, without any evidence of life-threatening injuries in the trauma primary survey, whereby complementary studies (portable chest X-ray, complete blood count) were requested. In the secondary survey, the previously described wound was located, and it was found not sucking, without subcutaneous emphysema. It was then sutured with interrupted 3/0 polypropylene stitches. Initial chest X-ray was reviewed and found to be within normal limits (see Figure 1(b)). The complete blood count presented leukocytosis and neutrophilia (leukocytes: 1642 x 10 ∧ 3/ul, neutrophils: 10.93 x 10 ∧ 3/ul) without any alteration in other cell lines, whereupon it was considered to continue clinical observation for 6 hours to proceed to a new assessment with an updated chest X-ray by the general surgery service. When the observation time was due, the patient was reassessed, presenting clinical signs of respiratory distress, with oxygen saturation of 88% with a FiO_2_ of 21% and poor pain control.

**Figure 2:**
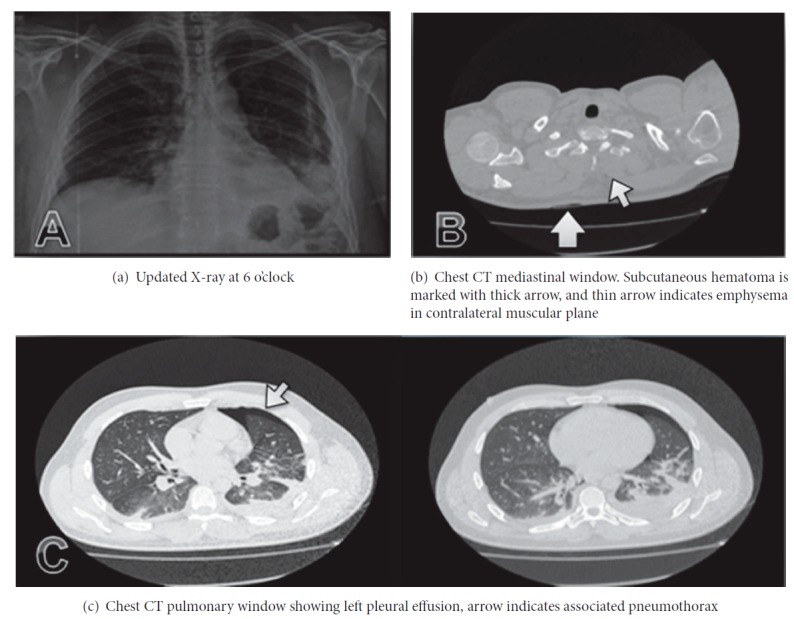


Chest X-ray was revised by the general surgery service, finding a left basal opacity (contralateral hemithorax to the stab wound) (see Figure 2(a)) without evidence of right pneumothorax or hemothorax (ipsilateral hemithorax to the stab wound), so assessment by the internal medicine service was requested. This last service contemplated pleural pathology and infectious diseases as differential diagnosis, so the realization of a chest computerized axial tomography scan was indicated. Subsequently, a joint assessment by emergency medicine and internal medicine services was held, where left pleural effusion was observed associated to haemopneumothorax in a contralateral location to the site of injury (see Figure 2(c)). Furthermore, subcutaneous hematoma was observed in right hemithorax, associated with emphysema in the left paravertebral muscle level, suggesting a likely oblique path of the wound directed from right to left and from cephalic to caudal (see Figure 2(b)).

Based on this consideration, the patient was once again assessed by the general surgery service who then performed a left closed thoracostomy as definitive treatment of traumatic hemopneumothorax. After the procedure, a total amount of 100cc of blood was collected, associated to air exit contained in the left hemithorax, confirming in this way the diagnosis of traumatic haemopneumothorax.

The subsequent evolution of the patient was satisfactory, presenting resolution of dyspnea, adequate pain relief with analgesics and after 4 days of incentive spirometer management, withdrawal of thoracostomy was decided and after that, hospital discharge. The Data of the Images, Laboratory data and Medical records used to support the findings of this study are included within the article.

## 3. Discussion

Traumatic pneumothorax is a frequent entity worldwide, which has reported an annual incidence from 18 to 28 for every 100,000 males and 1.2-6 per 100,000 women in the United States [9]. In our circle, there is a subregistry of trauma mechanisms and its secondary complications. However, in a study conducted in 2 hospitals of 2nd and 3rd level of a Colombian city (Cali-Valle del Cauca) it was determined that the stab wound represents the 10.6% as a mechanism of injury and represents the 5th cause of death by trauma constituting 4.1% [10].

Traumatic pneumothorax can be caused by a cutting or piercing weapon, penetrating projectiles, iatrogenesis, or blunt chest trauma [5]. Pneumothorax can be found as one of the most frequent complications of rib fractures. The latter can be seen in 40 to 50% of patients with chest trauma, and up to 51% of the patients may present an occult pneumothorax that was not observed in the initial chest X-ray; however, it can be found in the reassessment imaging studies or in a chest computerized axial tomography performed early [9].

Three ways of entry of air into the pleural cavity are described within the pathophysiology of pneumothorax: (A) communication between the alveolar spaces and the pleura, (B) the presence of organisms that produce gas in the pleural space, and (C) the direct or indirect communication between the atmosphere and the pleural space [11].

The presented case reported a patient with traumatic pneumothorax, contralateral to the site of a stab wound. Even though the entity is frequent in our circle, contralateral presentation to the injury is extremely uncommon. In major databases, there was not any documented similar case report; nevertheless, contralateral pneumothorax has been documented associated to pacemaker implantation [12], after pneumonectomy [13] or other pulmonary intervention surgical procedures [14].

The presentation of cases in which anatomical injuries are uncommon can lead to diagnostic errors and delays in an opportune treatment, taking into account that the clinical behavior of this entity can vary from the asymptomatic presentation to an acute situation that endangers life [6].

This entity is usually confirmed by diagnostic imaging (X-ray, ultrasound, or tomography). Nevertheless, chest radiographic studies can achieve a rate of false negatives up to 50% as in the reported case. Ultrasonography has a known role in the diagnosis of pneumothorax reaching a sensitivity of 95.3% and a specificity of 91%, identifying up to 63% of hidden pneumothorax cases [15]. However, chest tomography presents a near 100% sensitivity and a similar specificity for the diagnosis of pneumothorax; this is the reason why some studies suggest that chest CT is the gold standard for the diagnosis of this entity [16]. In our reported case, the addition of chest CT in order to confirm the presence of hemopneumothorax allowed inferring the oblique route of the stab wound through the findings of subcutaneous hematoma on the entry site and emphysema over the contralateral chest muscle tissue.

Nonetheless, early diagnosis of this entity requires knowledge of the circumstances that may give rise to its formation, a high degree of clinical suspicion and the appropriate request of imaging studies that confirm the initial diagnostic impression [17–19]

## 4. Conclusion

Wounds produced by piercing objects in the thorax are not frequently presented with injuries in unexpected anatomical regions, such as contralateral haemopneumothorax. Only a good physical examination, considering the injury mechanism, the attacking object, and later the imaging studies may help the physician to rule out life-threatening injuries during the primary and secondary survey of the trauma patient.

## Data Availability

The data of the images, laboratory data, and medical records used to support the findings of this study are included within the article.
